# Misinformation, Fears and Adherence to Preventive Measures during the Early Phase of COVID-19 Pandemic: A Cross-Sectional Study in Poland

**DOI:** 10.3390/ijerph182212266

**Published:** 2021-11-22

**Authors:** Bartosz M. Nowak, Cezary Miedziarek, Szymon Pełczyński, Piotr Rzymski

**Affiliations:** 1 Student Research Club of Clinical Immunology, Faculty of Medicine, Poznan University of Medical Sciences, 60-806 Poznań, Poland; poz.bartosznowak@gmail.com; 2 Student Research Group of Paediatric Surgery, Faculty of Medicine, Poznan University of Medical Sciences, 60-572 Poznań, Poland; 3 Student Research Group of Anaesthesiology and Intensive Therapy, Faculty of Medicine, Poznan University of Medical Sciences, 61-861 Poznań, Poland; szymon.pelczynski@gmail.com; 4 Department of Environmental Medicine, Poznan University of Medical Sciences, 60-806 Poznań, Poland; 5 Integrated Science Association (ISA), Universal Scientific Education and Research Network (USERN), 60-806 Poznań, Poland

**Keywords:** infodemic, pandemic, social media, fear, preventive measures, COVID-19, SARS-CoV-2

## Abstract

The response to the pandemic requires access to accurate information and public understanding and adherence to preventive measures. This online cross-sectional study of adult Poles (*n* = 1337) assessed the frequency of COVID-19 preventive behaviors, fears related to the COVID-19 pandemic, and beliefs in COVID-19-related conspiracy theories during the early phase of the COVID-19 pandemic when the nationwide lockdown was imposed (April 2020). As shown, 22% of surveyed admitted not to wash their hands frequently, while 12% did not use disinfectants. These two behaviors were also less frequent in individuals with medical education. The highest levels of pandemic-related fears were associated with health loss in relatives, pandemic-induced economic crisis, and government using a pandemic to control citizens by the state. A significant share of surveyed individuals believed that the pandemic was intentional action to weaken non-Chinese economies (32%) or was deliberately induced for profits from selling vaccines (27%). Men, individuals with no children, and subjects with lower education were significantly less likely to adhere to sanitary measures (handwashing, disinfection, avoiding face touching, changes in greeting etiquette, face-covering when coughing or sneezing), and were less concerned over self and relatives’ health. At the same time, men were less prone than women to the conspiracy theories related to the COVID-19 pandemic. The results indicate that adherence to sanitary measures during the pandemic can be a challenge also in developed countries, while misinformation campaigns (also concerning vaccines) have already affected the general public during the early phase of the epidemiological outbreak. The study provides observations that may be useful in the management of the public response to future epidemics.

## 1. Introduction

The COVID-19 pandemic has caused major economic and social disruptions and has overwhelmed the healthcare systems [1,2,3]. It has also been accompanied by the unprecedented scale of misinformation and flood of false news—the phenomenon for which the World Health Organization (WHO) has coined the term “infodemic” [4,5,6]. During the early phase of an outbreak, the data on SARS-CoV-2 and COVID-19 was limited, particularly regarding the persistence of SARS-CoV-2 on external surfaces, frequency of asymptomatic infections, the role of presymptomatic spread, potential treatment options, clinical features of the disease, and its broad clinical complications [7,8,9]. These knowledge gaps, along with a novel and not well-understood health threat, have led to high public anxiety, further amplified by the media coverage often using clickbait techniques and fear-promoting headlines and terms to generate a higher revenue [10,11]. In addition, the massive research output seen in the first months since the outbreak has confused journalists who were reporting the conflicting information and using preprints and pilot studies as definitive sources of scientific conclusions, selected politicians and celebrities were expressing opinions contradicting the scientific evidence, while the online social media were flooded with conspiracy theories and fake news [11,12]. The recommendations released by medical authorities such as WHO, Centers for Disease Control and Prevention in the United States, or European Centre for Disease Prevention and Control were often deliberately undermined, leading to a decrease in compliance with COVID-19 guidelines and favoring a spread of SARS-CoV-2 infections [13,14,15].

It is pivotal to learn the lesson from the infodemic during the early stage of the COVID-19 pandemic and understand its effects on awareness and knowledge in the general public. This is because it is plausible that similar outbreaks of viral zoonoses may take place in the future, particularly under increasing environmental degradation, urbanization, wild trade, and livestock farming [5,16,17]. As seen during the COVID-19 pandemic, broad access to online resources and platforms can be helpful in the rapid dissemination of pivotal scientific evidence [8,18], but may also be used to spread fake news—a phenomenon not faced at such a scale in the past [19,20,21]. Therefore, it is essential to study how different populations reacted to the COVID-19 pandemic in terms of adherence to safety measures, level of fear, and misinformation impacts.

The present cross-sectional study aimed to assess the awareness of COVID-19 preventive measures, the level of fears related to the COVID-19 pandemic, and beliefs in conspiracy theories associated with COVID-19 in the sample of adult Poles. The research was conducted in April 2020, during the first phase of the COVID-19 pandemic and during the nationwide lockdown. During this time, the number of confirmed infected and fatal cases in Poland increased from 7852 to 11,902 and from 286 to 562, respectively. The first COVID-19 case in the country was confirmed on 4 March 2020, the school and university closures were imposed on 11 March, and the borders were closed on 15 March, while a nationwide lockdown was imposed on 24 March and lasted until the beginning of May when nonessential shops, hotels, daycare centers, and kindergartens were reopened. The present study results reflect the time of limited information on COVID-19 and associated anxiety and fears over a novel and not well-understood health threat. Therefore, they give an overview of how the evidence-based information and unsupported claims affected the general public in Poland and provide an understanding of early reactions to the COVID-19 pandemic. Such observations may also be valuable for those who will be responsible for effective communication during the early stages of future crises, including those related to infectious diseases.

## 2. Materials and Methods

### 2.1. Survey

To assess the spread of misinformation in Poland, an anonymous, online survey based on a self-designed, structured questionnaire in the Polish language was designed (Supplementary Materials) and shared through online social media and message boards to induce a snowball effect. Such online research was previously recommended to reach the specific groups of interest while ensuring their safety under pandemic conditions [22]. The present survey was conducted between 15 and 27 April 2020, the period during which the strict nationwide lockdown was imposed. The inclusion criteria for the study were Polish nationality and ≥18 years old.

The questionnaire employed in the study aimed to assess the following:

1. Level of adherence to various COVID-19 preventive measures: frequent hand washing, avoiding face touching, avoiding handshaking, covering mouth when coughing or sneezing, and using disinfection liquids. Face masking was not evaluated as using face masks were recommended in Poland for the first time when this study was initiated.
2. Level of fears related to the pandemic and its implications: (i) job loss, (ii) loss of one’s health due to COVID-19, (iii) loss of relatives’ health due to COVID-19, (iv) pandemic-induced economic crisis, (v) pandemic-induced political crisis, (vii) use of pandemic to control citizens by the government. The level of each fear was evaluated with 10-point Likert-type scales, where 1—no fear, 5—a medium level of fear, and 10—very high level of fear.
3. Frequency of beliefs in circulating conspiracy theories on COVID-19: (i) pandemic induced by 5G network, (ii) pandemic induced by the Chinese government to weaken other economies, (iii) pandemic induced to weaken Chinese economy, and (iv) pandemic induced for profits of pharmaceutical companies from selling vaccines. At the time of the study (April 2020), there was no data on the prevalence of each pandemic-related conspiracy theory in Poland. Therefore, these four conspiracy theories were selected based on the experience of co-authors as they were among those most frequently encountered.
4. The internal consistency reliability of scales used to evaluate fears related to the pandemic was determined with Cronbach’s alpha and showed acceptable reliability of α = 0.79.
5. The demographic data on each surveyed individual included age, gender, place of living (urban or rural), level of education (primary, secondary, tertiary, or vocational), and whether those surveyed had children.

### 2.2. Statistical Analysis

The statistical analyses were conducted using Statistica v.13.1 (StatSoft Inc., Tulsa, OK, USA). The frequencies of adherence to sanitary measures, levels of fears related to the COVID-19 pandemic, and popularity of conspiracy theories related to COVID-19 were compared between women and men, urban and rural inhabitants, individuals with tertiary and other levels of education, individuals with medical and nonmedical education, and individuals having and not having children. Mann–Whitney U-test was used to test the differences in scores given using Likert-type scales. The frequencies were compared with Pearson’s χ^2^ test. A *p*-value < 0.05 was considered statistically significant.

## 3. Results

### 3.1. Demographic Characteristics

The survey was completed by 1380 individuals, of which 43 were aged <18 years old (3.1%) and were therefore excluded. Overall, 1337 eligible questionnaires were used in the analysis. Considering that the population targeted by the study was approximately 31.5 million individuals (number of adults aged 18 or more [23]), a power calculation indicated that this size of the sample gives a margin error of 2.7% at the confidence level of 95%. The demographic breakdown of the studied population is summarized in Table 1. The mean ± SD age of the studied individuals was 35.1 ± 11.1. Most of the surveyed individuals were women (84.9%), inhabited urban areas (67.8%), and had higher education (53.7%).

### 3.2. Adherence to COVID-19 Preventive Measures

In general, frequent hand washing was reported by 77.6% of studied individuals, avoiding face touching by 73.3%, avoiding handshaking by 91.4%, covering mouth when coughing by 82.8%, while the use of the disinfectant was declared by 88.4%. A significant difference in adherence to preventive measures was found between various subgroups (Table 2). Women were more likely to display all considered behaviors, decreasing the risk of contracting SARS-CoV-2. Higher adherence to the majority of them was seen in individuals having children. Having a tertiary education was associated with better adherence only to selected behaviors. Individuals with medical education revealed a significantly lower frequency of handwashing, covering the mouth when coughing or sneezing, and using disinfection. No differences in this regard were found between inhabitants of rural and urban areas were found (Table 2).

### 3.3. Level of Fears Related to COVID-19 Pandemic

Studied individuals demonstrated varying fear levels regarding different aspects of the COVID-19 pandemic and its potential consequences. The fear over the health of relatives was the highest (median, interquartile range = 9, 7–10) followed by fear of pandemic-induced economic crisis (8, 6–10) and using pandemic to control the citizens (8, 5–10), fear over own health (6, 3–9), pandemic-induced political crisis (6, 3–9), and fear of losing a job (4, 1–8). Statistical analysis revealed several differences in fear levels. Women displayed greater fear over every considered aspect. Individuals with children had a higher fear of job loss, fear over their own health and health of family members, and fear related to the pandemic-induced economic crisis. Tertiary and medical educations were also found to be associated with a higher level of fear over selected aspects associated with the COVID-19 pandemic (Table 3).

### 3.4. Beliefs in Conspiracy Theories

The majority of studied individuals did not trust in any considered conspiracy theory regarding the COVID-19 pandemic. However, 31.5% of surveyed admitted to believing that the Chinese government intentionally induced the COVID-19 pandemic to weaken other economies, while 27.2% believed the pandemic was induced so pharmaceutical companies could benefit from vaccines roll-out. A minority of surveyed individuals believed that the COVID-19 pandemic was a deliberate action to weaken the Chinese economy (16.8%) or that its origins are associated with the development of the 5G network (14.5%). All of these conspiracy theories were more frequently believed by women. Individuals with children, those inhabiting rural areas, and having education other than tertiary were more susceptible to selected conspiracy theories (Table 4).

## 4. Discussion

The present study explored the knowledge on COVID-19 and adherence to preventive measures in adult Poles during the first phase of the pandemic in 2020. Considering that novel epidemics are plausible in the future [16,24,25], it is pivotal to learn from observations from the COVID-19 outbreak, understand the public’s early reactions, and identify groups requiring additional efforts to communicate essential information regarding safety measures. In addition, the results of the present study provide a reference point for similar investigations conducted during later phases of the COVID-19 pandemic.

Strikingly, as many as 22.5% of all surveyed individuals and 30.5% of those with medical education admitted not to washing their hands frequently during the pandemic’s early phase, demonstrating that the adherence to the most basic sanitary measures may be challenging not only in low-income regions but also in developed countries such as Poland. This is even more disturbing if one considers that over 10% did not use liquid disinfectants, which were already widely available at that time. As shown, women paid more attention to handwashing than men. Several studies conducted before the pandemic indicated that women wash their hands more often and have better knowledge about hand hygiene in the general population [26,27,28] and healthcare professionals [29]. Investigations conducted during the COVID-19 pandemic correspond to the present results, indicating that women are better educated regarding hand hygiene [30,31]. At the same time, men are more likely to underestimate the role of this behavior [32]. Another study conducted at the beginning of the COVID-19 pandemic in Poland indicated that women, in general, were more likely to present an involved attitude manifested, inter alia, by following the recommendations more closely [33], and this was also confirmed in the present survey.

All in all, this highlights that information campaigns during the response to respiratory disease epidemics should pay additional attention to target male populations. The present study also demonstrated that women and individuals having children more frequently avoided touching their faces. During the early pandemic phase, such behavior was recommended as a protection measure as the hand-to-face contact increases the risk of infection and spread of various pathogens [34]. Nevertheless, the more recent studies suggest that this transmission route is not as important as was initially assumed, especially if hand disinfectants are widely available [35,36]. The present study indicated that women, individuals with children, and individuals with higher education were more likely to use disinfectants. The present study also demonstrated that women, individuals with higher education, and individuals with children more frequently avoided handshaking. It is known that replacing the handshake with, e.g., a fist or elbow bump may reduce the transmission of infectious agents [37], which is particularly relevant in populations such as Polish, in which the handshake is the most popular and traditional form of greeting. As observed, women and individuals having children were also more careful about covering their faces during coughing. The cough etiquette, including covering the mouth and nose during coughing or sneezing, is a recommended element of respiratory hygiene [38], although this does not fully block the transmission of infectious respiratory diseases [39].

In summary, being a woman, followed by having children, was the most important factor determining the positive changes in hygiene habits during the early phase of the COVID-19 pandemic. The place of living—rural or urban—was not found to play any role in this regard.

It is known that novel and not well-understood epidemiological threats can cause an increased level of fear, anxiety, and stress in the general public. Studies conducted at the beginning of the COVID-19 pandemic demonstrated that Poles revealed higher levels of perceived stress compared to populations of various other countries, including other European Union member states [40]. The present study explores the specific types of fear related to the COVID-19 pandemic. As observed, women and individuals with children were more afraid to lose their job. Interestingly, the data from different highly developed and developing countries suggest that during the COVID-19 pandemic, men were more susceptible to losing their job [41,42]. Other studies indicated that both women and men were experiencing nearly identical rates of employment loss [43]. The present research did not indicate that people without higher education were more afraid to lose their job than their better-educated counterparts, although it is known that lower-skilled workers are more prone to lose their job [44]. In general, unemployment and anxiety associated with job instability during an unexpected pandemic should be given special attention because it may lead to a higher frequency of mental issues [45]. A topic that is highly connected with the fear of unemployment is a risk of a potential economic crisis. The present study identified that women, individuals with lower education, and those having children were more afraid of economic crisis due to the COVID-19 pandemic. Economic crisis nearly always leads to the rise of unemployment, particularly among women and unskilled workers [44,46]. In turn, fear of economic crisis among people with children is related to the feeling of responsibility for life and the wellbeing of relatives. However, anxiety and fear among parents can potentially adversely affect the children and thus may also adversely influence their future [47]. Higher fear of economic crisis may also be due to the perception that it may be of a unique kind, further exaggerating the anxiety [48]. As shown, at the beginning of the COVID-19 pandemic in Poland, women were more concerned not only about losing their life due to COVID-19, but also the life of a family member. In general, during a health crisis such as a pandemic, the fear of death is often higher [49]. Cross-sectional studies conducted in other populations, e.g., Cuban or Chinese, have revealed that women and parents are more prone to various fears related to COVID-19 [50,51,52]. In general, there is substantial evidence that women report higher levels of fear due to the number of gender differences [53]. Various observations highlight that women may be more fearful of novelty, including novel technologies, medicines and vaccines, and novel epidemiological threats [54,55,56].

Moreover, the present study analyzed the frequency of believing in different conspiracy theories related to the COVID-19 pandemic. As shown, over 30% believed that the pandemic was intentionally induced to weaken various economies. The other Polish study conducted in a similar period (April 2020) showed that approximately one-third of Poles revealed a full agreement with at least one conspiracy theory related to the COVID-19 pandemic, the majority of which concerned the government conspiracies [57]. The present study also indicated that over 25% of those surveyed believed that the pandemic was induced to gain profits from vaccine roll-out. This finding highlights that misinformation on the potential COVID-19 vaccines may start long before any vaccine candidates were authorized for use. The other Polish study conducted at the beginning of the pandemic indicated that over 10% of surveyed Poles believed that SARS-CoV-2 was created by pharmaceutical companies [57]. Moreover, the analysis of Facebook users’ comments posted on the selected Polish media profiles in November and December 2020 indicated that 85% of content related to the COVID-19 vaccines was negative with numerous conspiracy theories such as that the vaccines were created only for the profit of pharmaceutical companies or that the vaccines were already prepared before the pandemic [58]. This may also explain, at least partially, the high level of vaccine hesitancy seen in the Polish population right before and after the authorization of the first COVID-19 vaccines [59,60]. The first polls, conducted in November 2020, demonstrated that only 20% of Poles declared a willingness to vaccinate, a figure that increased to 36% in December 2020 and to 55% in mid-February 2021 [61,62]. However, at the beginning of October 2021, the share of Poles who had received at least one COVID-19 vaccine dose reached 52%.

As shown, women, individuals with other than tertiary education, and those having children were more prone to believe that the COVID-19 pandemic was related to the development of the 5G network. Several studies analyzed the spread of misinformation about COVID-19 and 5G conspiracy theories in social media. The study conducted by Bruns et al. [63] distinguished five chronological phases of the worldwide spread of such fake news on Facebook. The present research was conducted after the last distinguished phase by authors (29 March to mid-April 2020) [63]. The results indicate that conspiracy theory related to the 5G network was already circulating and absorbed by some individuals in the Polish population. Another study examining COVID-19 and 5G conspiracy theories on Twitter indicated the importance of the possibility of reporting fake news on social media to reduce the spread of misinformation [64].

Women and individuals with children also more frequently believed that the pandemic is associated with the pharmaceutical companies’ efforts to popularize vaccines and generate income from their sales. The idea that the pandemic is a pretext to mass vaccination programs was supported by the anti-vaccine movements [65]. The first cases of SARS-CoV-2 infection were reported in Wuhan, China; therefore, the Chinese government was suspected to be responsible for spreading the virus [66]. In addition, more women and people with lower education believed that the pandemic could be an instrument to weaken the Chinese economy or action conducted by the Chinese government to weaken other economies. The latter opinion might have been reflected in the increased incidence of ethical discrimination, social stigma, and Sinophobia [67]. The anti-Asian sentiments related to COVID-19 were reported in Poland even before the first case of the COVID-19 was identified in the country [68].

It can be hypothesized that the women and individuals having children surveyed in the present study were in a more precarious state at the beginning of the COVID-19 pandemic in Poland. These groups not only revealed higher fear of different aspects of the COVID-19 pandemic and better adherence to preventive measures but were also more prone to conspiracy theories on predatory motivations behind the pandemic, i.e., intentionally induced for one’s profit. Interestingly though, various prepandemic studies found, with very few exceptions, no significant differences in conspiracy thinking between women and men [69,70,71,72]. This highlights that the COVID-19 pandemic may represent an extraordinary situation in terms of conspiracy beliefs. However, contrary to the present findings, the US study conducted in a similar pandemic period (April 2020) found that women were significantly less likely than men to endorse COVID-19 conspiracy theories, regardless of their partisanship [73]. All in all, this indicates that gender differences in COVID-19 conspiracy beliefs may exist, but these gender differences may vary across different populations due to potential sociocultural, psychological, and educational factors that would require further studies.

Study limitations should be stressed. Firstly, the research was based on an anonymous online survey, while the adherence to sanitary measures was declarative. This excludes the possibility of verifying the data on the more objective ground. Secondly, the studied sample could not be considered as a representative for the entire Polish population, and potential extrapolations should be made with appropriate acknowledgment. Thirdly, the subset of men was underrepresented, which is often the case in voluntary survey investigations [74,75]. Moreover, an online survey may attract the attention and willingness of younger and better-educated individuals, which is also seen in the present study. On the other hand, the younger and better-educated part of the population may play an important role in response to the pandemic via engagement as the volunteer medical workforce, delivering food and sanitation, speaking out for a more vulnerable group, and communicating accurate information through online social media [76,77].

## 5. Conclusions

The present study documented the adherence to sanitary measures, the level of fears related to the COVID-19 pandemic, and the frequency of beliefs in conspiracy theories during the early phase of the COVID-19 pandemic in Poland. As shown, surveyed Poles feared most for relatives’ health, although a worrisome share (including individuals with medical education) did not apply to recommendations to wash hands more frequently, while beliefs that the pandemic was induced by Chinese government or for the benefit of pharmaceutical companies selling vaccines were relatively frequent. Some important gender differences were observed. Although women were more frequently adhering to different sanitary measures, they had greater fears related to the pandemic and were more prone to conspiracy theories. The results suggest that effective management of pandemics requires counteracting fake news and conspiracy theories as soon as possible and identification and targeting of specific groups to increase adherence to pivotal sanitary recommendations.

## Figures and Tables

**Table 1:** The demographic breakdown of the studied group (*n* = 1337).

Characteristics	Women (*n* = 1135)	Men (*n* = 202)	Total (*n* = 1337)
**Age** (mean ± SD)	35.8 ± 10.9	30.8 ± 11.5	35.1 ± 11.1
**Place of living** (% (n)) Urban area Rural area	67.0 (760) 33.3 (375)	72.3 (146) 27.7 (56)	67.8 (906) 32.2 (431)
**Education** (% (n)) Primary Vocational Secondary education (nonmedical) Secondary education (medical) During nonmedical studies During medical studies Higher education (nonmedical) Higher education (medical)	1.4 (16) 3.9 (44) 24.5 (278) 2.3 (26) 8.6 (98) 3.8 (43) 51.6 (586) 3.9 (44)	5.0 (10) 6.4 (13) 20.3 (41) 0 (0) 19.8 (40) 5.0 (10) 40.1 (81) 3.5 (7)	1.9 (26) 4.3 (57) 23.9 (319) 1.9 (26) 10.3 (138) 4.0 (53) 49.9 (667) 3.8 (51)
**Having children** (% (n))	63.6 (722)	29.7 (60)	58.5 (782)

**Table 2:** The adherence (%) to preventive measures in different subgroups of the studied population (*n* = 1337). *p*-value indicating significant differences is given in bold (Pearson’s χ^2^ test).

Behavior	Men / Women	Rural / Urban	Tertiary Education / Other Education	Medical Education / Nonmedical Education	Children No Children
Frequent hand washing	71.8/78.7 **0.03**	77.0/77.9 >0.05	78.6/76.6 >0.05	70.0/78.5 **0.03**	77.0/78.7 >0.05
Avoiding face touching	62.9/75.2 <**0.001**	71.9/74.0 >0.05	74.0/72.5 >0.05	73.1/73.3 >0.05	76.6/68.7 **0.001**
Avoiding handshake	85.2/92.5 <**0.001**	91.2/91.6 >0.05	94.0/88.4 <**0.001**	90.8/91.5 >0.05	93.6/88.3 <**0.001**
Covering mouth when coughingor sneezing	70.3/85.0 <**0.001**	84.7/81.9 >0.05	83.3/82.3 >0.05	75.4/83.6 **0.02**	85.0/79.6 **0.01**
Using disinfection	80.2/89.9 <**0.001**	89.1/88.1 >0.05	90.4/86.1 **0.02**	78.5/89.5 <**0.001**	90.2/86.0 **0.02**

**Table 3:** The fears ((median (interquartile range)) related to the COVID-19 pandemic and its consequences in different subgroups of the studied population (*n* = 1337), evaluated using a 10-point Likert-type scale. *p*-value indicating significant differences is given in bold (Mann–Whitney U-test).

Fear	Men / Women	Rural / Urban	Tertiary Education / Other Education	Medical Education / Nonmedical Education	Children / No Children
Job loss	3 (1–7)/4 (1–8) <**0.001**	3 (1–8)/4 (1–8) >0.05	4 (1–8)/3 (1–8) >0.05	1.5 (1–6)/4 (1–8) <**0.001**	4 (1–9)/3 (1–7) **0.006**
Fear overown health	4 (2–6)/7 (4–10) <**0.001**	6 (3–9)/6 (3–9) >0.05	7 (4–9)/5 (3–9) <**0.001**	5 (3–8)/6 (3–9) **0.01**	8 (5–10)/5 (3–8) <**0.001**
Fear over family member health	7 (5–9)/10 (7–10) <**0.001**	9 (6–10) /10 (7–10) >0.05	9.5 (7–10)/9 (6–10) **0.04**	9 (7–10)/9 (7–10) >0.05	10 (7–10)/9 (6–10) <**0.001**
Economic crisis	8 (5–9)/9 (7–10) <**0.001**	8 (6–10)/9 (7–10) >0.05	9 (7–10)/8 (5–10) <**0.001**	8 (6–10)/9 (6–10) >0.05	9 (7–10)/8 (6–9) <**0.001**
Political crisis	5 (3–8)/6 (3–9) **0.02**	6 (3–9)/6 (3–9) >0.05	7 (4–9)/5 (2–8) <**0.001**	6 (3–8)/6 (3–9) >0.05	6 (3–9)/6 (3–8) >0.05
Increased control of the citizens	8 (4–10)/9 (5–10) **0.01**	8 (5–10)/9 (5–10) **0.02**	9 (6–10) /8 (4–10) >0.05	8 (4–9)/9 (5–10) **0.004**	9 (6–10)/8 (5–10) <**0.001**

**Table 4:** The frequency (%) of beliefs in conspiracy theories on COVID-19 in different subgroups of the studied population (*n* = 1337). *p*-value indicating significant differences is given in bold (Pearson’s χ2 test).

Conspiracy Theory	Men / Women	Rural / Urban	Tertiary Education / Other Education	Medical Education /Nonmedical Education	Children /No Children
COVID-19 pandemic induced by 5G network	5.5/16.1 <**0.001**	16.7/13.5 >0.05	12.1/17.3 **0.008**	6.2/15.4 **0.005**	19.3/7.8 <**0.001**
COVID-19 pandemic as an action to weaken the Chinese economy	10.9/17.9 **0.01**	21.4/14.7 **0.002**	14.2/19.9 **0.006**	12.3/17.3 >0.05	18.4/14.6 >0.05
COVID-19 pandemic induced by the Chinese government to weaken other economies	25.2/32.6 **0.04**	35.7/29.5 **0.02**	28.7/34.7 **0.02**	27.7/31.9 >0.05	34.8/26.9 **0.002**
COVID-19 pandemic as an action of pharmaceutical industry to sell vaccines	19.3/28.6 **0.006**	29.0/26.4 >0.05	25.8/28.9 >0.05	16.9/28.3 **0.006**	31.2/21.6 <**0.001**

## Supplementary Materials

The following are available online at www.mdpi.com/article/10.3390/ijerph182212266/s1.
