# Supplementary material for: Misinformation, Fears and Adherence to Preventive Measures during the Early Phase of COVID-19 Pandemic: A Cross-Sectional Study in Poland
Source: Int J Environ Res Public Health. 2021 Nov 22;18(22):12266. doi: 10.3390/ijerph182212266 (PMC8618240; doi:10.3390/ijerph182212266)
Supplement: Supplementary file 1 [file ijerph-18-12266-s001.zip › ijerph-1430523-supplementary.pdf]

Supplementary material for

" Misinformation, fears and adherence to preventive measures during the early phase of COVID-19 pandemic: A cross-sectional study in Poland"

Bartosz M. Nowak, Cezary Miedziarek, Szymon Pełczyński, Piotr Rzymski

Translated Questionnaire

Demographic data

Your gender:

- Woman
- Man
- Other

Your age (in years): ......

Your level of education:

- Primary education
- Vocational education
- Secondary education (non-medical)
- Secondary education (medical)
- During higher education (non-medical)
- During higher education (medical)
- Higher education (non-medical)
- Higher education (medical)

Place of living, where you spend most of your life:

- Village
- City up to 25 000 inhabitants
- A city with 25 000 and 100 000 inhabitants
- A city with 100 000 and 200 000 inhabitants
- A city with 200 000 and 500 000 inhabitants
- A city with over 500 000 inhabitants

Do you have any children?

- Yes
- No

Adherence to preventive measures

Please indicate whether you adhere to the following preventive measures recommended during the COVID-19 pandemic:

- **Frequent hand washing**
  - Yes
  - No
- **Avoiding face touching**
  - Yes
  - No
- **Avoiding handshake**
  - Yes
  - No
- **Covering mouth when coughing or sneezing**
  - Yes
  - No
- **Using disinfection**
  - Yes
  - No

Fears related to COVID-19 pandemics

Please assess your level of fear related to the following COVID-19 pandemic aspects
(Scale 1 to 10, where 1- no fear, 5 - a medium level of fear, 10 - very high level of fear)

Fear type	Your level of fear									
	1	2	3	4	5	6	7	8	9	10
Fear over job loss										
Fear over the loss of one's health due to COVID-19										
Fear over the loss of relatives' health due to COVID-19										
Fear over pandemic induced economic crisis										
Fear over pandemic induced political crisis										
Fear over the use of the pandemic to control citizens by the government										

Conspiracy theories on COVID-19

Please indicate whether you believe that the COVID-19 pandemic was induced by the 5G network?

- Yes
- No

Please indicate whether you believe that the COVID-19 pandemic was induced by the Chinese government to weaken other economies?

- Yes
- No

Please indicate whether you believe that the COVID-19 pandemic was induced to weaken the Chinese economy?

- Yes
- No

Please indicate whether you believe that the COVID-19 pandemic was induced for profits of pharmaceutical companies from selling vaccines

- Yes
- No
